# TRIM26 promotes non-small cell lung cancer survival by inducing PBX1 degradation

**DOI:** 10.7150/ijbs.81726

**Published:** 2023-05-29

**Authors:** Yuening Sun, Peng Lin, Xiumin Zhou, Ying Ren, Yuanming He, Jingpei Liang, Zhigang Zhu, Xiaofeng Xu, Xinliang Mao

**Affiliations:** 1 Guangdong Institute of Cardiovascular Diseases, Guangdong Key Laboratory of Vascular Diseases, the Second Affiliated Hospital of Guangzhou Medical University, Guangzhou, 511436, P. R. China.; 2 Guangdong Key Laboratory of Protein Modification and Degradation, School of Basic Medical Sciences, Guangzhou Medical University, Guangzhou, 511436, P. R. China.; 3 Department of Pharmacology, College of Pharmaceutical Sciences, Soochow University, Suzhou, Jiangsu, 215123, P. R. China.; 4 Department of Oncology, The First Affiliated Hospital of Soochow University, Suzhou, P. R. China.; 5 Department of Biology, GMU-GIBH Joint School of Life Sciences, The Guangdong-Hong Kong-Macau Joint Laboratory for Cell Fate Regulation, Guangzhou Medical University, 511436, P. R. China.; 6 Division of Hematology & Oncology, Department of Geriatrics, Guangzhou First People's Hospital, College of Medicine, South China University of Technology, Guangzhou, Guangdong 510180, P. R. China.; 7 Department of Urology, Jinling Hospital of Nanjing University, Nanjing, 210093, P. R. China

**Keywords:** Non-small cell lung cancer, Pre-B cell leukemia transcription factor 1, Tripartite motif containing 26

## Abstract

The transcription factor PBX1 is regarded as an oncogene in various cancers, but its role in non-small cell lung cancer (NSCLC) and the detailed mechanism is not known. In the present study, we found that PBX1 is downregulated in NSCLC tissues and inhibits NSCLC cell proliferation and migration. Subsequently, we performed an affinity purification-coupled tandem mass spectrometry (MS/MS) and found the ubiquitin ligase TRIM26 in the PBX1 immunoprecipitates. Moreover, TRIM26 binds to and mediates PBX1 for K48-linked polyubiquitination and proteasomal degradation. Noticeably, TRIM26 activity depends on its C-terminal RING domain when it is deleted TRIM26 loses its function towards PBX1. TRIM26 further inhibits PBX1 transcriptional activity and downregulates the PBX1 downstream genes, such as RNF6. Moreover, we found that overexpression of TRIM26 significantly promotes NSCLC proliferation, colony formation, and migration in contradiction to PBX1. TRIM26 is highly expressed in NSCLC tissues and predicts poor prognosis. Lastly, the growth NSCLC xenografts is promoted by overexpression of TRIM26 but is suppressed by TRIM26 knockout. In conclusion, TRIM26 is a ubiquitin ligase of PBX1 and it promotes while PBX1 inhibits NSCLC tumor growth. TRIM26 might be a novel therapeutic target for the treatment of NSCLC.

## Introduction

Lung cancer is the most frequent cause of cancer-related deaths worldwide, accounting for an estimated 1.6 million deaths a year, 85% of which are non-small cell lung cancers^1^. NSCLC is a molecularly heterogeneous disease and understanding its biology is crucial for the development of targeted therapies^2^. Tyrosine kinase inhibitors and immunotherapy targeting epidermal growth factor receptor (EGFR) have made significant progress in the treatment of NSCLC^3^, however, the overall remission and survival rates for patients with NSCLC remain very low, especially in developing countries. Therefore, it is urgent to explore the molecular process in the pathophysiology of NSCLC and find novel potential targets for the treatment of NSCLC.

PBX1 (Pre-B cell leukemia transcription factor 1) is a member of the TALE (three amino acid loop extension) class of homeodomain transcription factors, which is related to the occurrence and development of many cancers, including those derived from stomach^4^, ovary^5^, blood^6^, and prostate cells^7^, however, the role of PBX1 in NSCLC is not well known. Moreover, PBX1 protein has been found to be degraded via the ubiquitin proteasome pathway and it can be stabilized by the deubiquitinase USP9x^8^. Inhibition of USP9x leads to PBX1 degradation and sensitizes prostate cancer cells to chemotherapeutics^8^. It is known that protein ubiquitination is mediated by a cascade of enzymes, including ubiquitin activating enzyme, ubiquitin conjugating enzymes and ubiquitin ligases. However, the ubiquitin ligase of PBX1 remains elusive.

In the present study, tripartite motif-containing 26 (TRIM26), a member of the TRIM family of ubiquitin ligases, is identified as a ubiquitin ligase of PBX1 during the characterization of the PBX1 interactome by affinity purification-coupled tandem mass spectrometry (AP-MS/MS). TRIM26 induces PBX1 degradation therefore inhibiting its transcriptional activity, and promoting NSCLC cell proliferation and xenograft growth.

## Materials and Methods

### Primary NSCLC tissues

Samples of confirmed NSCLC patients were provided by the First Affiliated Hospital of Soochow University. ​Informed consent was obtained before collecting the samples for the study. The research in this work followed the principles of Helsinki Declaration and was approved by the Review Committee and ethics Committee of Soochow University.

### Cell lines

Human embryonic kidney cells (HEK293T) were kindly provided by Dr. Michael F. Moran, University of Toronto, Canada. NSCLC cell lines were purchased from Procell Life Science &Technology Co., Ltd. (Wuhan, China).

### Plasmids and antibodies

PBX1, RNF6 and Ub were cloned as described previously^6^,^9^. TRIM26 was cloned from HEK293T cells by RT-PCR using primers 5'-atttgcggccgcATGGATTTAGAGAAAAATTA-3' and 5'-ccgctcgagTCAGTGACGGTCATAGGCAG-3'. The full-length cDNA was then inserted into a pcDNA3.1 vector with an HA, Flag or Myc tag. Monoclonal anti-HA, anti-Myc, and anti-Flag were obtained from Medical & Biological Laboratories Co., Ltd. (Nagoya, Japan). Antibodies against TRIM26, RNF6 and GAPDH were purchased from Proteintech (Wuhan, China). Antibodies against PBX1, K48-Ub, and K63-Ub were purchased from Cell Signaling Technology, Inc., (Danvers, MA, USA). An antibody against Ub was purchased from Santa Cruz Biotechnology (Santa Cruz, CA, USA). HRP-labeled goat anti-mouse and goat anti-rabbit IgG (H+L) antibodies were purchased from Beyotime Institute of Biotechnology (Nantong, China).

### Affinity purification-coupled mass spectrometry

Cells expressing Flag-PBX1 were treated with MG132 (10 μM) for 4 hrs followed by lysis as described previously^10^. After clarification, 10 mg proteins were subjected to incubate with Protein A+G beads for 1 hr at room temperature. After removing the beads, the supernatants were further incubated with anti-Flag beads overnight at 4 °C. Subsequent procedures were described previously^11^. After strict washes, the beads were then added 2 x SDS loading buffer and boiled for SDS-PAGE. The gels were then cut into two slices for each sample and the gels were then subjected to protein extract. The detailed methods and MS data analysis were described as previously^11^. Proteins with more than 2 unique peptides identified with a confidence > 95% were chosen for further studies.

### Small interfering RNAs (siRNA)and short hairpin RNA (shRNA)

The specific siRNAs and shRNAs were synthesized by Ribobio (Guangzhou, Guangdong, China) and were transfected into HEK293T or NSCLC cells using polyethylenimine (PEI, Sigma) or Lipofectamine™ 2000 (Invitrogen, USA) as the gene carrier.

### CRISPR genome editing

The optimal sgRNA target sequences were designed using the Benchling CRISPR Genome Engineering tool (https://www.benchling.com). sgRNA target sequences were cloned into lentiCRISPRv2 as reported previously^12^. sgTRIM26 #1: 5'-GAGCGGCTGAAGGTGGACAA-3', sgTRIM26 #3: 5'-GGACCAGAGGCAGTACATTGGC-3'.

### Immunoblotting (IB)

After transfection with appropriate plasmids, cells were lysed in a lysis buffer followed by SDS-PAGE and IB as described previously^10^.

### Immunoprecipitation (IP)

After transfection with appropriate plasmids for 48 hrs, total cell lysates (TCL) were prepared and subjected to IP as described previously^10^.

### Immunofluorescence (IF) assay

Cells were first fixed in 4% paraformaldehyde, permeabilized with 0.02% Triton X-100, and then blocked with PBS supplemented with 20% bovine serum. Cells were incubated with anti-TRIM26 and anti-PBX1 specific antibodies, which were diluted at 1:100 in PBS with 0.3% Tween-20 and 1% BSA and incubated overnight at 4 °C. Following 3 washes (5 min per wash) at room temperature with PBS, cells were incubated with secondary antibodies mixture containing goat anti-mouse IgG labeled with fluorescein isothiocyanate (1:200; FITC; Beyotime, Haimen, China) and goat anti-rabbit IgG labeled with cyanine (Cy)3 (1:200; Beyotime) at room temperature for 3 hrs. Images were observed by laser scanning confocal microscope.

### Cell viability assay

Cell viability was measured by MTT assay (Beyotime) as described previously^6^.

### Cell Proliferation by EdU staining

NSCLC cells transfected with appropriate plasmids were incorporated with EdU, followed by staining with Apollo® fluorescent dyes (Cell-Light™ EdU Apollo ® 567 *In vitro* Kit, Ribobio, Guangzhou, China) according to the manufacturer's instruction, then analyzed on a fluorescence microscopy as described previously^13^.

### Cell cycle analysis by flow cytometry

NSCLC cells transfected with appropriate plasmids were collected for cell cycle regents staining followed the instruction from the manufacturer (MultiSciences Biotech Co., Ltd, Hangzhou, China) and subjected to analysis on a BD flow cytometer as described previously^14^.

### Cycloheximide (CHX) chase assay

After transfected with plasmids of interest for 36 hrs, NSCLC cells were treated with CHX (100 μg/ml, Sigma-Aldrich) for 0 to 12 hrs before being collected for protein preparation. Total proteins were fractionated on SDS-PAGE before IB analyses with specific antibodies as described previously^14^.

### Reverse transcription-quantitative polymerase chain reaction (RT-qPCR)

The RT-qPCR primers for TRIM26 were: 5'-TCCAGGACCAGAGGCAGTA-3'(forward) and 5'-TCTTCCGTGGATACCTGTTTA-3' (reverse). The specific primers for PBX1, RNF6 and GAPDH and the RT-qPCR protocol were described previously^13^. Data quantification was determined by the 2^-ΔΔCt^ method.

### Xenograft model

Nude Balb/c mice from Guangdong Medical Laboratory Animal Center (female, 5-week-old), were bred at the animal facility of Guangzhou Medical University. A549 and H226 cells infected with lentiviral TRIM26 or sgTRIM26 were injected subcutaneously into the flanks of mice at a density of 5 × 10^6^ cells per injection (n = 6). Mice and tumors were continuously monitored for 15 days on a daily base. At the endpoint, mice were sacrificed, and tumor xenografts were then removed, weighed and stored for further applications. ​All experiments were conducted in accordance with the relevant guidelines and regulations of animal welfare. All animal studies were conducted with the approval of the Institutional Animal Care and Use Committee of Guangzhou Medical University.

### Immunohistochemical (IHC) staining

Formalin-fixed xenografts were embedded in paraffin and sectioned using standard techniques. MaxVision^TM^reagent (MXB^TM^ Biotechnologies, Fuzhou, China) was applied to each slide as instructed by the manufacturer. The slides were then treated with 0.05% diaminobenzidine and 0.03% H_2_O_2_ in 50 mM Tris-HCl, pH 7.6, and finally counterstained with hematoxylin. A negative control was also included for each xenograft specimen by substituting the primary antibody with pre-immunization serum.

### Densitometric analysis

Densitometric analysis of immunoblots in the protein stability was executed as described previously using ImageJ© software developed by the National Institutes of Health^6^.

### Statistics

All experiments were executed at least three times, and the results were expressed as mean ± SD where applicable. GraphPad Prism V8.0 (GraphPad® Software, San Diego, CA) was used for statistical analysis. Difference of multiple groups was using one-way ANOVA followed by Tukey's test or Newman-Kueuls test. Value of *p* < 0.05 was determined statistically significant.

## Results

### PBX1 is downregulated in NSCLC tissues and inhibits NSCLC cell proliferation and migration

**Figure 1:**
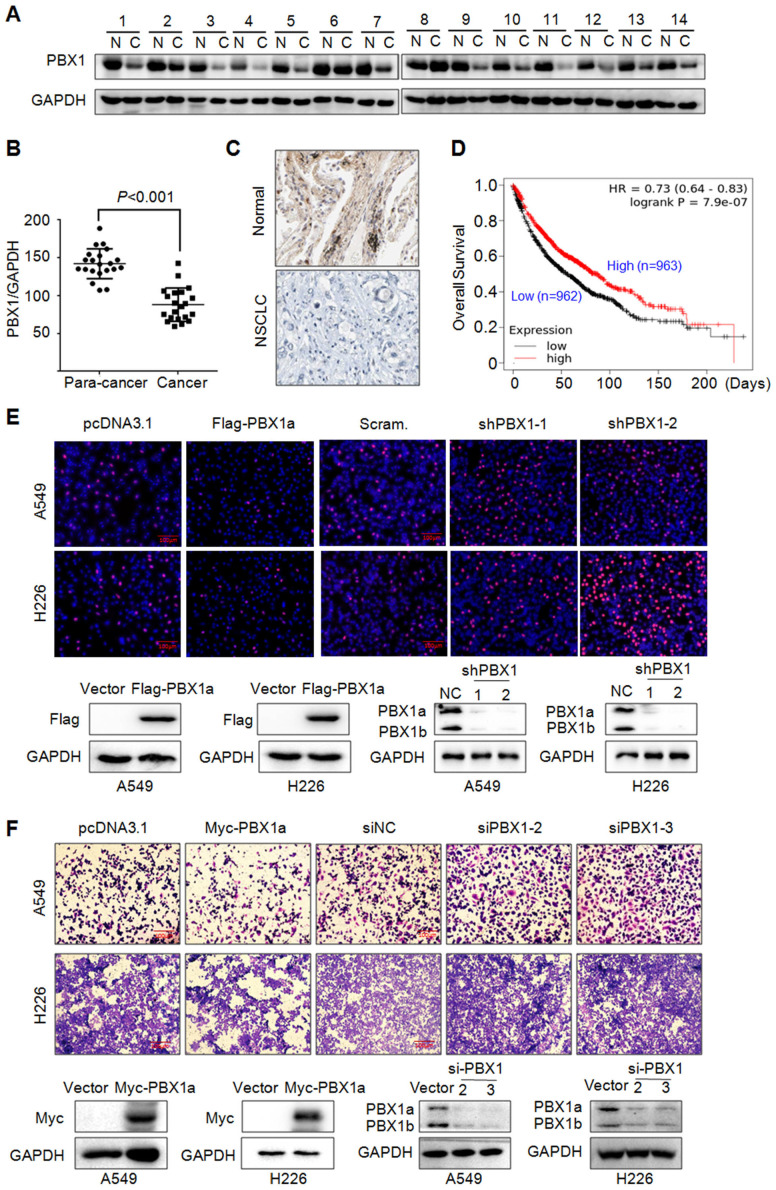
** PBX1 is downregulated in NSCLC and inhibits NSCLC cell proliferation and migration.** (A) NSCLC (C) and adjacent normal (N) tissues were collected for IB assays. (B) Statistical analyses of PBX1 in NSCLC tissues. (C) Representative analysis of PBX1 expression in NSCLC by immunohistochemical analysis. (D) Overall survival analysis of NSCLC patients in terms of PBX1 expression. Data was retrieved from Kaplan-Meier Plotter. (E-F) A549 and H226 cell lines were overexpressed with PBX1 or were knocked down for PBX1. Cells were then subjected to EdU staining (E) or transwell assays to evaluate cell migration (F).

PBX1 has been reported to promote tumorigenesis in various cancers, but its role in NSCLC is not well understood. To this end, we first evaluated the expression level of PBX1 in NSCLC and its adjacent tissues. To our surprise, PBX1 was downregulated in NSCLC tissues compared to individual paired control (Fig. 1A) and this difference was striking (Fig. 1B). This finding was confirmed by IHC (Fig. 1C). Interestingly, PBX1 was positively and significantly associated with the overall survival of NSCLC patients based on the analysis of a large population with NSCLC. The higher the PBX1 expression, the higher the overall survival (Fig. 1D). This finding indicated that PBX1 might inhibit NSCLC cell proliferation and survival. To confirm this hypothesis, PBX1 was overexpressed into or knocked down from typical NSCLC cell lines A549 and H226, followed by EdU corporation assay and migration assays. EdU or 5-ethynyl-2'-deoxyuridine is an analog of uridine, that can be incorporated into newly synthesized DNA therefore indicating cell proliferation. Our study found that when PBX1 was overexpressed, the EdU positive cells were markedly reduced, in contrast, more cells were positive for EdU staining when PBX1 was knocked down (Fig. 1E). Consistent with finding, similar results were found in the migration study. The portion of the migrated cells was negatively associated with PBX1 expression (Fig. 1F). All these studies thus indicated that PBX1 displays anti-NSCLC activity by inhibiting NSCLC cell proliferation and migration.

### TRIM26 interacts with PBX1

**Figure 2:**
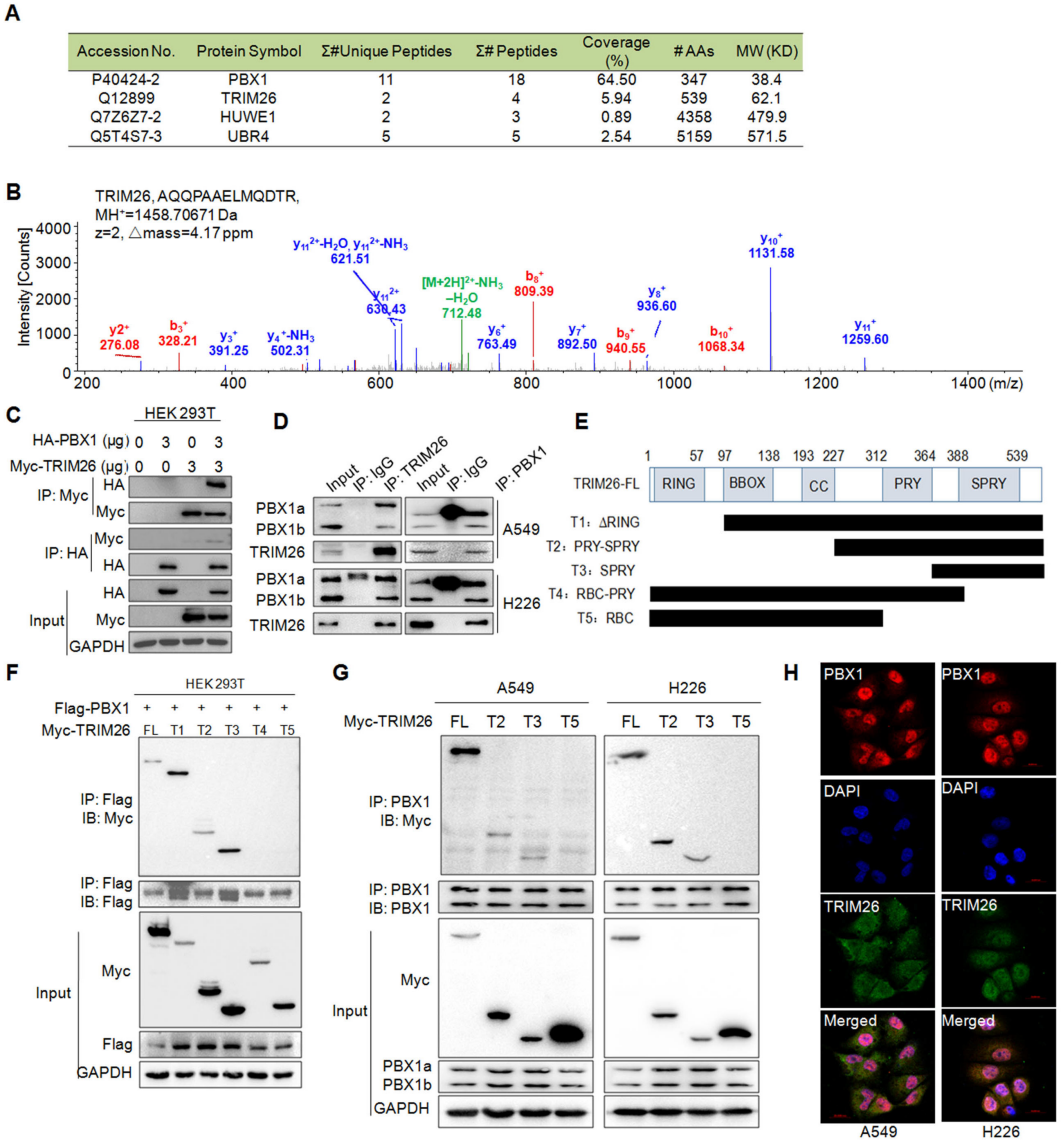
** TRIM26 interacts with PBX1 protein.** (A) The coverage and unique peptides of PBX1 and TRIM26 in the affinity-purified PBX1-interacting proteins by tandem mass spectrometry (MS/MS). (B) A representative MS/MS spectrum of TRIM26. The b- and y-ions were annotated and the peptide sequence, charge state (z), MH+, and Δmass were shown. (C) HA-PBX1 and Myc-TRIM26 were co-transfected into HEK293T cells for 48 hrs, then cells lysates were subjected to IP and IB assays. (D) Cell lysates were subjected to IP/IB with specific TRIM26 or PBX1 antibody or IgG, followed by IB assays. (E) Schematic diagram of TRIM26 truncates. (F) TRIM26 and its truncates were co-transfected with Flag-PBX1 into HEK293T cells for 48 hrs, followed by cell lysate preparation and IP/IB assays.(G) TRIM26 and its truncates were transfected into A549 and H226 cells for 48 hrs, followed by cell lysate preparation and IP/IB assays.(H) A549 and H226 cells were immunostained with specific antibodies against PBX1 (red) and TRIM26 (green), and then counterstained with DAPI (blue) to show nuclei.

Given PBX1 is processed via the ubiquitin-proteasome pathway^8^, we wondered to know the ubiquitin ligase mediating PBX1 ubiquitination. To this end, we performed an AP-MS/MS assay to find out proteins interacting with PBX1, from which several possible E3 ligases including UBR4, HUWE1, and TRIM26 were identified in the PBX1-interacting proteins (Fig. 2A). By comparing their unique peptides, the coverage, and molecular weight as well as probable binding specificity (HUWE1 was also found in our other AP-MS assays)^10^, TRIM26 was chosen for further investigation. The representative MS spectrum of TRIM26 was shown in Fig. 2B. The interaction between TRIM26 and PBX1 was further confirmed in both HEK293T and NSCLC cell lines by reciprocal IP/IB assays (Fig. 2C and D). To further identify the specific binding region of TRIM26 interacting with PBX1, we constructed five truncates of TRIM26 based on its domains (Fig. 2E). The subsequent co-transfection and IP/IB assays in HEK293T cells demonstrated that all the truncates containing the SPRY domain could bind to PBX1, in contrast, those truncates lacking the SPRY domain failed to interact with PBX1 (Fig. 2F). These findings were also confirmed in NSCLC cells (Fig. 2G). Therefore, TRIM26 binds to PBX1 via its SPRY domain. To better understand the physical interaction between TRIM26 and PBX1, an IF assay was performed in NSCLC cells. The result showed that TRIM26 and PBX1 were co-localized to nuclei although TRIM26 was also found in cytosol (Fig. 2H). Taken together, these studies indicate that TRIM26 binds to PBX1 through the SPRY domain.

### TRIM26 directs PBX1 for K48-linked polyubiquitination

**Figure 3:**
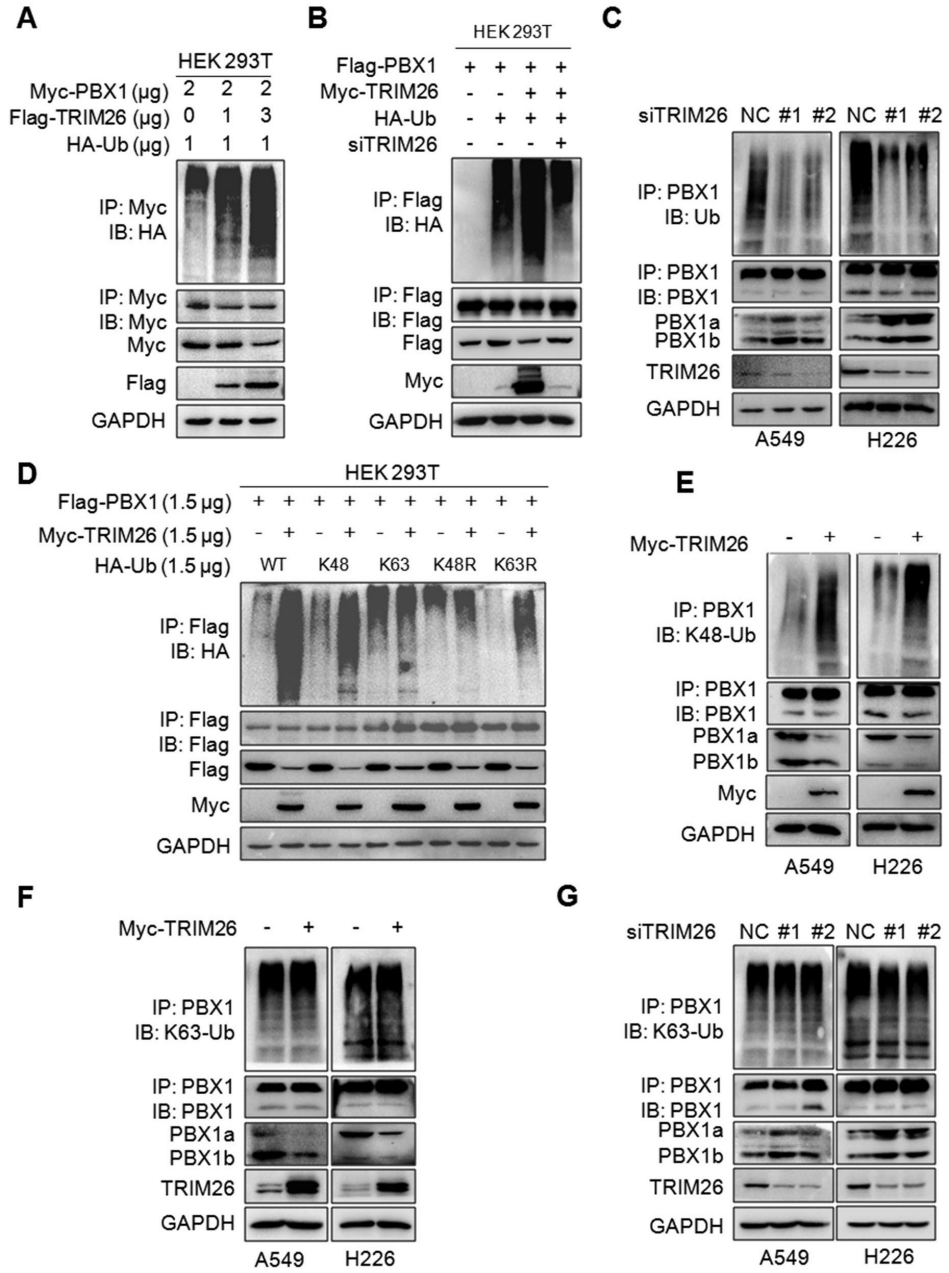
** TRIM26 mediates PBX1 K48-linked ubiquitination.** (A) Myc-PBX1, Flag-TRIM26 and HA-Ub plasmids were co-transfected into HEK293T cells followed by IP/IB assay as indicated. (B) Flag-PBX1, Myc-TRIM26, siTRIM26 and HA-Ub plasmids were co-transfected into HEK293T cells followed by IP/IB assay as indicated. (C) A549 and H226 cells were transfected with siRNA, 48hrs later cell lysates were subjected to IP/IB assays as indicated. (D) A Myc-TRIM26 plasmid was co-transfected with Flag-PBX1, HA-K48-Ub, HA-K63-Ub, HA-K48R, or HA-K63R into HEK293T cells followed by IP/IB assay as indicated. (E-F) A549 and H226 cells were transfected with TRIM26 plasmids for 48 hrs, followed by IP/IB assays as indicated. (G) A549 and H226 cells were transfected with siTRIM26 for 48 hrs, followed by IP/IB assays as indicated.

Given TRIM26 is a ubiquitin ligase, we speculated whether it could mediate the ubiquitination of PBX1. To this end, Myc-PBX1, Flag-TRIM26 and HA-Ub plasmids were co-transfected into HEK293T cells, the subsequent IP/IB assays showed that TRIM26 promoted PBX1 polyubiquitination in a dose-dependent manner (Fig. 3A). In contrast, the ubiquitination was partly abolished when TRIM26 was knocked down (Fig. 3B). These results were also confirmed in NSCLC cells in which TRIM26 knockdown directly reduced the polyubiquitination level (Fig. 3C). Given K48- and K63-linked ubiquitination are two typical ubiquitination forms, we co-transfected HEK293T cells with TRIM26 and K48-, K48R-, K63- and K63R-Ub plasmids, respectively, the subsequent IP/IB assays showed that PBX1 was heavily ubiquitinated only in the presence of K48- and K63R-Ub but not K48R- and K63-Ub, suggesting that TRIM26 might mediate PBX1 ubiquitination in a K48- but not K63-linked manner (Fig. 3D), which was further verified in NSCLC cells by a K48-Ub and a K63-Ub specific antibody (Fig. 3E, F and G). Taken together, TRIM26 mediates PBX1 ubiquitination with the K48-linked manner.

### TRIM26 promotes PBX1 degradation in proteasomes

**Figure 4:**
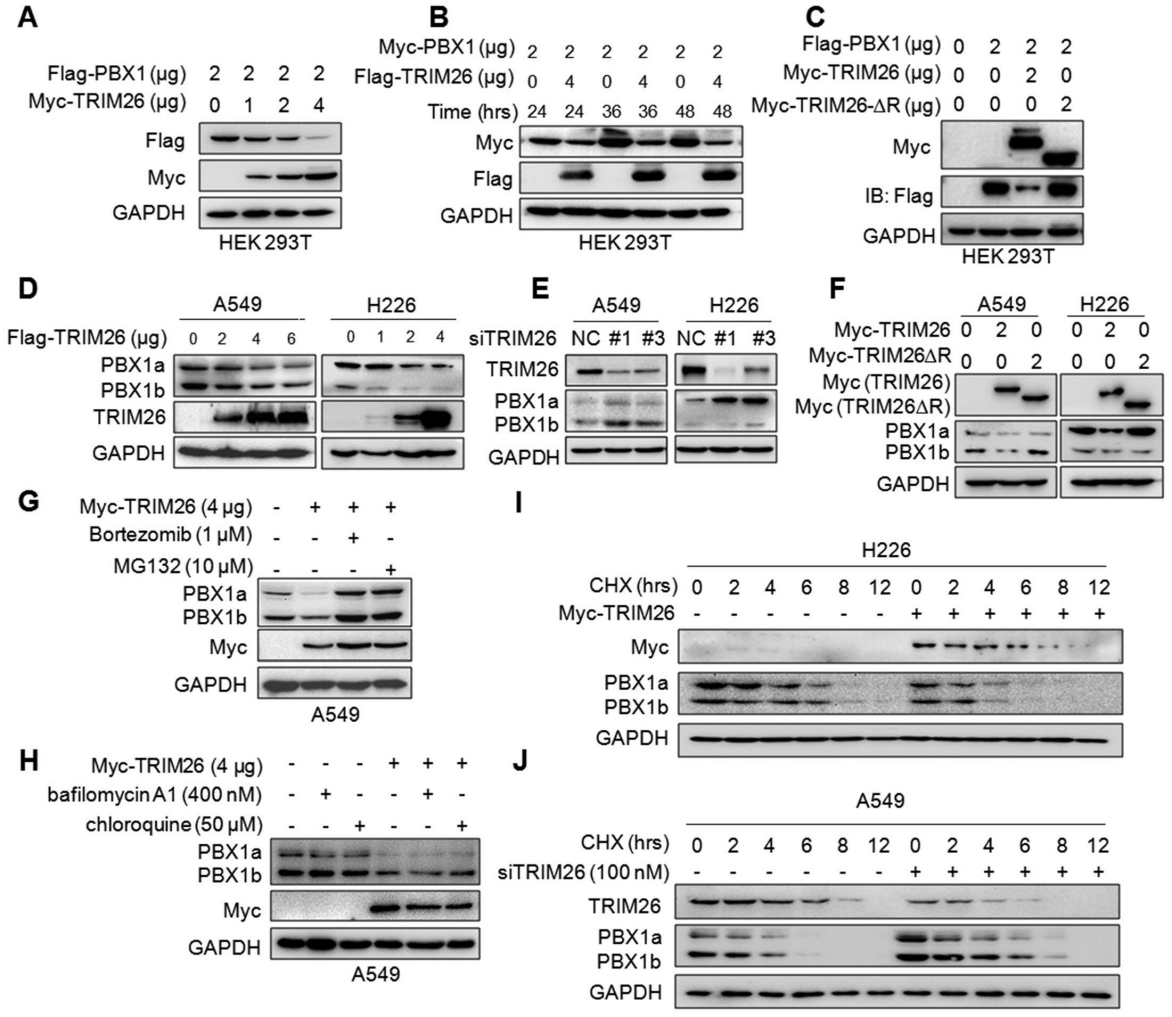
** TRIM26 promotes PBX1 degradation in proteasomes.** (A) Flag-PBX1 and increased Myc-TRIM26 plasmids were co-transfected into HEK293T cells for 48 hrs, and then cell lysates were subjected to immunoblotting assay. (B) Myc-PBX1 and Flag-TRIM26 plasmids were co-transfected into HEK293T cells for the indicated periods followed by immunoblotting assay. (C) Flag-PBX1, Myc-TRIM26 or Myc-TRIM26-∆R plasmids were co-transfected into HEK293T cells for 48 hrs, and then cell lysates were subjected to immunoblotting assay. (D) Flag-TRIM26 plasmids were transfected into A549 or H226 cells for 48 hrs, and then cell lysates were subjected to immunoblotting assay. (E) siTRIM26 sequences were transfected into A549 and H226 cells for 48 hrs, followed by cell lysate preparation and IB assays as indicated. (F) Myc-TRIM26 or Myc-TRIM26-∆R plasmids were transfected into A549 or H226 cells for 48 hrs, and then cell lysates were subjected to immunoblotting assay. (G, H) A549 cells were transfected with TRIM26 plasmids for 48 hrs, followed by the treatment with proteasome inhibitors (G) or lysosome inhibitors (H) treatment followed by immunoblotting assay. (I) A549 cells were subjected to transfection of siTRIM26, (J) H226 cells were transfected with TRIM26 plasmids, 36 hrs later cells were incubated with cycloheximide (CHX) for indicated periods before being collected for IB assays.

The above results indicated that TRIM26 promotes PBX1 for K48-linked polyubiquitination, which is a hallmark of protein degradation. Therefore, we wondered whether PBX1 protein could be degraded by TRIM26. To this end, the Flag-PBX1 and Myc-TRIM26 plasmids were co-transfected into HEK293T cells, and IB assays showed that PBX1 protein was downregulated by TRIM26 in a dose- and time-dependent manners (Fig. 4A and B). Furthermore, when the RING domain, the critical domain responsible for E3 ligase activity, was deleted, TRIM26 lost its enzymatic activity therefore failing to downregulate PBX1 (Fig. 4C), suggesting that TRIM26 degraded PBX1 through its ubiquitin ligase activity. This hypothesis was further confirmed in NSCLC cell lines A549 and H226, in which overexpression of TRIM26 downregulated PBX1 (Fig. 4D) while knockdown of TRIM26 stabilized PBX1 (Fig. 4E). Moreover, consistent with the study in HEK293T cells, TRIM26-∆R inactivated TRIM26 and failed to downregulate the PBX1protein in both NSCLC cell lines (Fig. 4F). Furthermore, the proteasomal inhibitors (MG132 and bortezomib, BZ) but not lysosomal inhibitors (bafilomycin A1 and chloroquine) could abolish PBX1 degradation induced by TRIM26 (Fig. 4G and H). To confirm this conclusion, we measured the effects of TRIM26 on the stability of PBX1 protein by CHX chase assays. As shown in Fig. 4I, overexpression of TRIM26 significantly decreased the half-life of PBX1 protein. In contrast, knockdown of TRIM26 could delay the rate of PBX1 degradation (Fig. 4J). All the above results thus collectively demonstrated TRIM26 promotes PBX1 degradation in proteasomes after K48-linked polyubiquitination.

### TRIM26 suppresses PBX1 transcriptional activity

**Figure 5:**
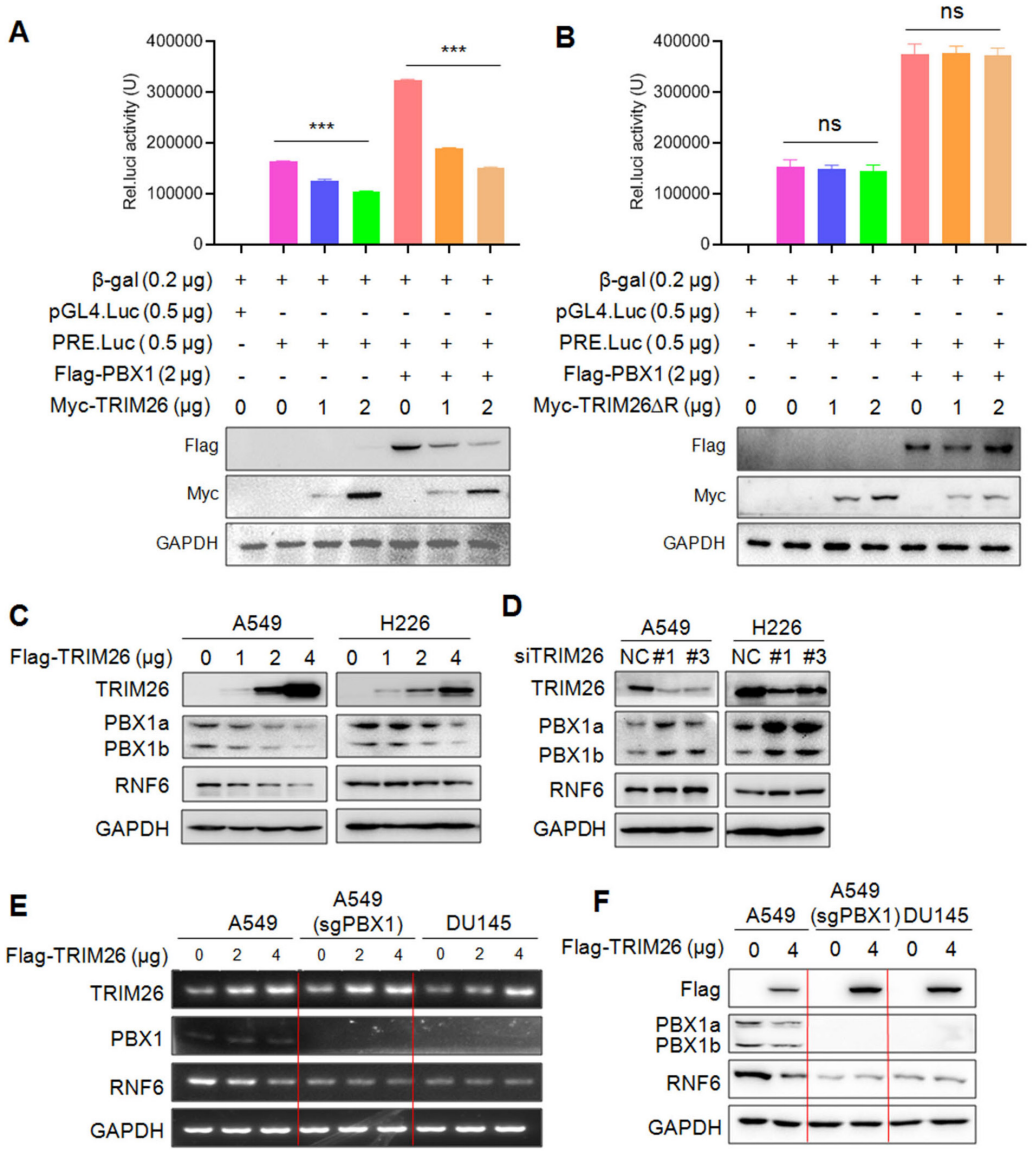
** TRIM26 suppresses PBX1 transcriptional activity.** (A-B) HEK293T cells were co-transfected with Flag-PBX1, PRE.Luci, β-gal and TRIM26 (A) or TRIM26∆R (B) plasmids for 48 hrs, cell lysates were prepared for luciferase activity measurement and IB assays. mean±SD (n=3). ***, *P*<0.001; ns, not significant. (C-D) NSCLC cell lines were transfected with TRIM26 plasmids (C) or siTRIM26 (D) for 48 hrs, followed by IB assays to evaluate the expression levels of PBX1 and its downstream gene RNF6. (E-F) PBX1 were knocked out from A549 by its specific sgRNA. Cells were then transfected with a TRIM26 plasmid for 48 hrs. Total RNA was extracted for RT-PCR against TRIM26, PBX1, RNF6 or GAPDH (E). Cell lysates were subjected to IB against specific proteins as indicated (F). DU145 lacking PBX1 was used as a control.

As a transcription factor of the TALE family, PBX1 regulates transcription of downstream target genes through binding to its specific recognition element at the promoter region^6^. Given that TRIM26 downregulates PBX1, we wondered whether TRIM26 suppressed its transcriptional activity. To this end, HEK293T cells were co-transfected with TRIM26, PBX1, and its recognition element driving-luciferase (PRE.Luci) plasmids^6^, followed by luciferase assay. The results revealed that introduction of PBX1 promoted the luciferase activity, but it was markedly decreased by TRIM26 (Fig. 5A). Notably, the suppressive activity of TRIM26 depended on its RING domain, when the N-terminal RING domain was deleted, TRIM26 lost its activity towards PBX1 transcriptional activity (Fig. 5B), which was consistent with the ΔRING TRIM26 on PBX1 stability (Fig. 4C and F). To confirm this finding, we further measured the protein level of RNF6, a known downstream gene modulated by PBX1^6^, in NSCLC cells upon PBX1 overexpression or knockdown. The subsequent assays showed that overexpression of TRIM26 downregulated RNF6 protein along with degrading PBX1 in NSCLC cells (Fig. 5C).

In contrast, RNF6 was upregulated when TRIM26 was knocked down in NSCLC cells (Fig. 5D). To find out whether TRIM26-downrgulated RNF6 expression was PBX1 dependent, A549 cells were knocked out PBX1 followed by TRIM26 overexpression, the subsequent mRNA and protein measurement showed that TRIM26 downregulated RNF6 at both mRNA and protein levels (Fig. 5E and F), but when PBX1 was knocked out, TRIM26 failed to alter RNF6 expression (Fig. 5E and F). Moreover, we applied DU145 cells that do not express PBX1^8^ as a PBX1-knockout control. Because DU145 does not express PBX1, overexpression of TRIM26 failed to alter RNF6 expression at either the mRNA or the protein levels (Fig. 5E and F). Therefore, these assays confirmed that TRIM26 suppressed transcriptional activity of PBX1 and inhibited the expression of its downstream target genes.

### TRIM26 inhibits NSCLC cell proliferation, migration and invasion

**Figure 6:**
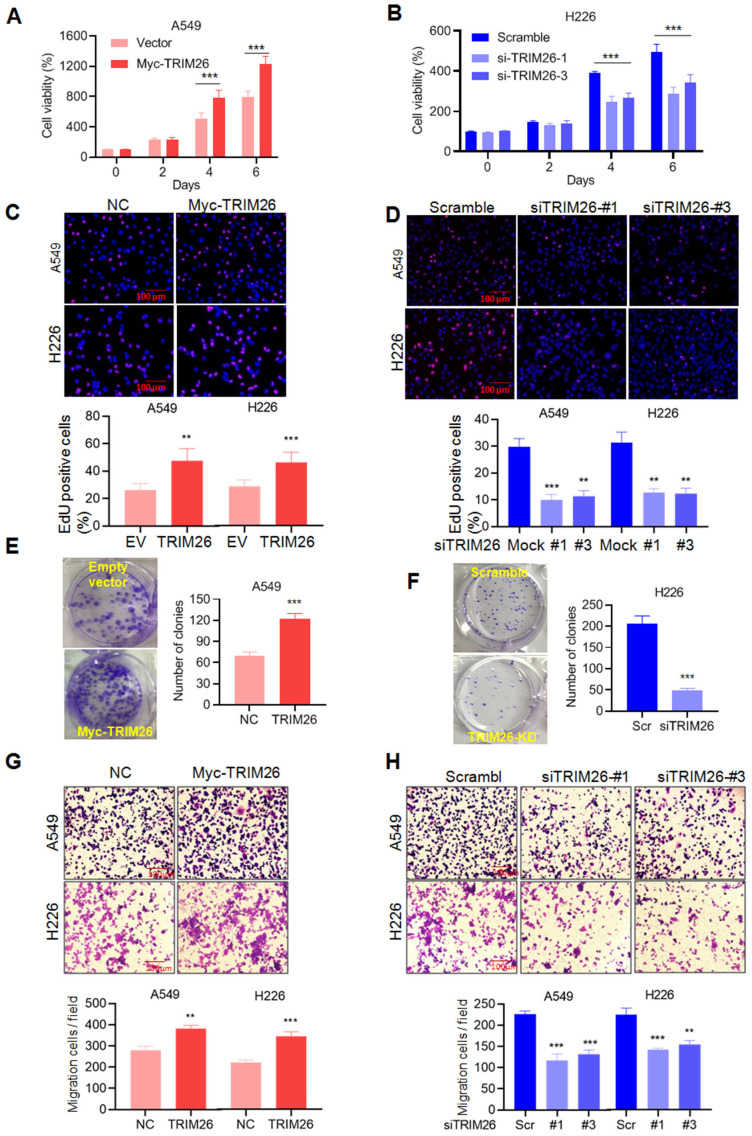
** TRIM26 promotes NSCLC cell proliferation and migration in association with PBX1 degradation.** (A, B) A549 cells were transfected with a TRIM26 plasmid while H226 cells were transfected with siTRIM26, followed cell proliferation assessment by MTT assay. mean±SD (n=3). ***, *P*<0.001, versus control. (C, D) Cell proliferation was evaluated by EdU incorporation assay in A549 and H226 cells after transfection of TRIM26 plasmids or siTRIM26. mean±SD (n=3). **, *P*< 0.01, ***, *P*< 0.001, versus control. (E, F) A549 cells were transfected with TRIM26 plasmids and H226 were transfected with siTRIM26, followed by colony formation assay. Compared with control, ***, *P*<0.001. (G, H) A549 and H226 cells were transfected with Myc-TRIM26 or siTRIM26 for 48 hrs, followed by transwell assays. mean±SD (n=3). **, *P*< 0.01, ***, *P*< 0.001, versus control.

Given PBX1 is found to inhibit NSCLC cell proliferation and migration (Fig. 1), we wondered the effects of TRIM26 on NSCLC. To this end, we measured the effects on NSCLC in terms of cell proliferation and metastasis by a series of analyses including MTT, EdU incorporation, colony formation and transwell assays. The results showed that overexpression of TRIM26 promoted NSCLC cell proliferation (Fig. 6A and C), colony formation (Fig. 6E) and migration/invasion (Fig. 6G). In contrast, TRIM26 knockdown suppressed cell proliferation (Fig. 6B and D), reduced colony formation (Fig. 6F) and migration/invasion (Fig. 6H). Therefore, TRIM26 is highly expressed in NSCLC cells and promotes NSCLC cell proliferation, survival and migration/invasion.

**Figure 7:**
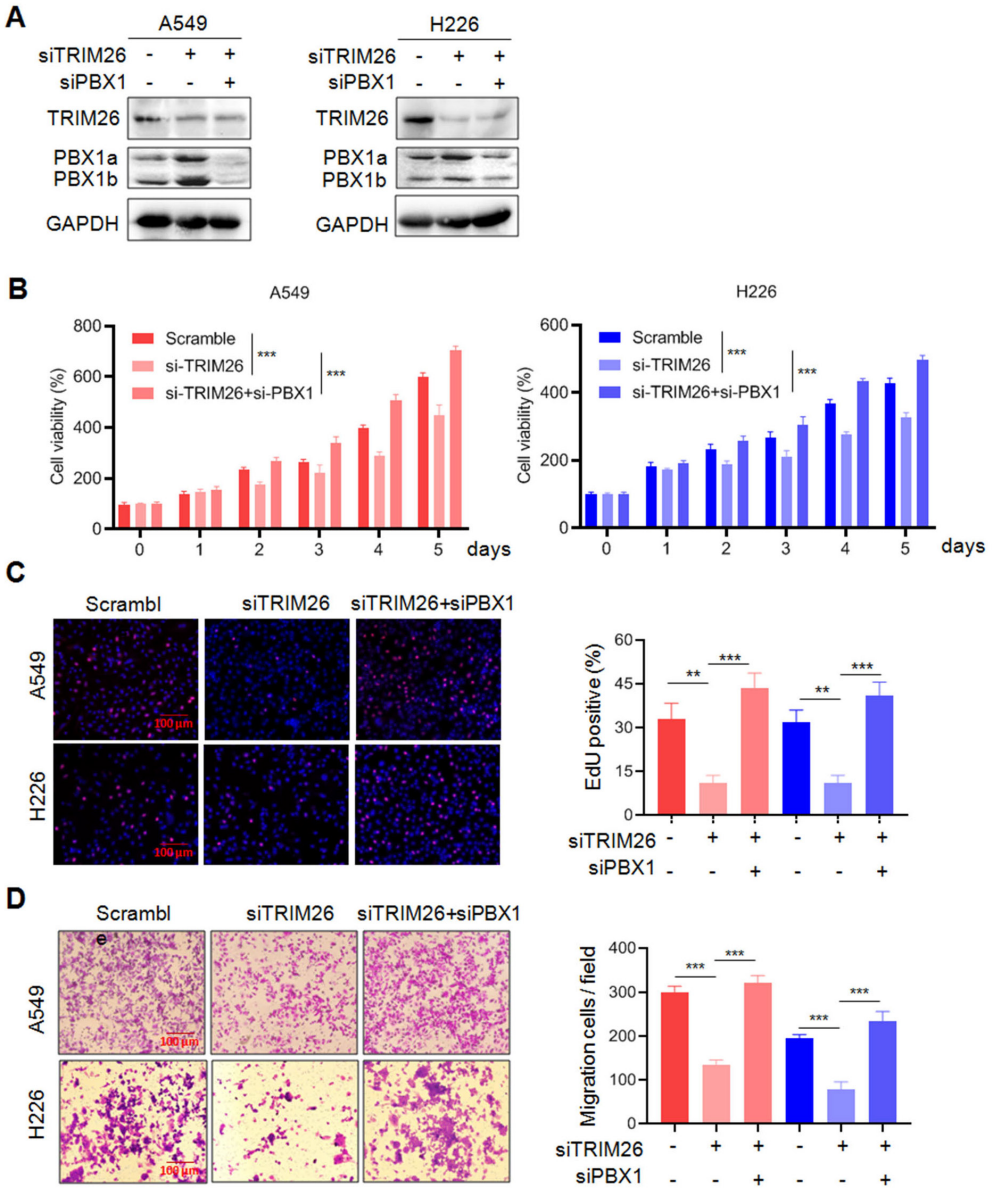
** Knockdown of PBX1 partly abolishes the effects of TRIM26 knockdown on NSCLC cell proliferation, survival and migration.** (A) A549 and H226 cells were transfected with siTRIM26 alone or co-transfected with siPBX1, for 24 hrs, followed cell lysate preparation and Western blot assays. B-D, Cell viability (B), EdUincorporation assay (C) and transwell assays (D) were performed in A549 and H226 cells after transfection with siTRIM26 alone or co-transfection with siPBX1. Mean±SD (n=3). **, *P*< 0.01, ***, *P*< 0.001, versus control.

To find out whether TRIM26 acts on NSCLC cells via PBX1, we examined the effects of PBX1 knockdown on TRIM26 activity. As shown in Fig. 7A, knockdown TRIM26 increased PBX1 protein, but it was downregulated when PBX1 was knocked down. The subsequent function assays showed that knockdown of TRIM26 led to decreased cell survival (Fig. 7B), proliferation (EdU staining, Fig. 7C) and migration (Fig. 7D), but all these effects were rescued by PBX1 knockdown (Fig. 7B-D). Therefore, these findings further confirmed that TRIM26 regulates NSCLC cell proliferation, survival and migration via PBX1 at least in part.

### TRIM26 is overexpressed in NSCLC tissues and promotes NSCLC tumor growth in nude mice

**Figure 8:**
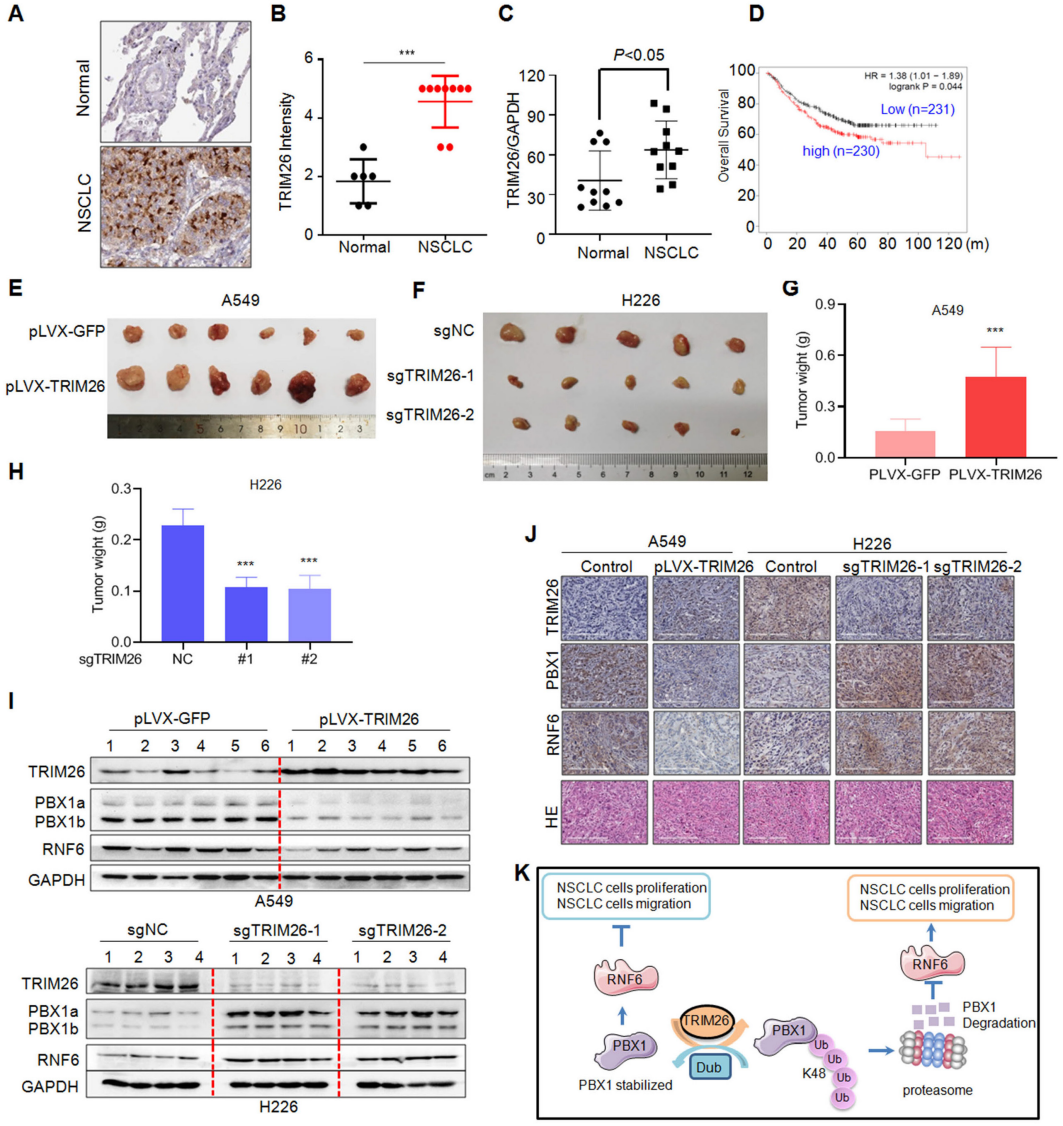
** TRIM26 promotes NSCLC tumor growth in nude mice by downregulating PBX1.** (A) Representative NSCLC and their para-cancerous tissues were collected for IHC assay against TRIM26 (A). (B) The statistical analyses on TRIM26 in the IHC assays as shown in A. (C) NSCLC and the paired normal tissues were subjected to IB, the ratio of TRIM26/GAPDH were analyzed. (D) TRIM26 expression is negatively associated with overall survival of patients with NSCLC. (E) A549 cells infected with TRIM26 or GFP lentivirus were injected into nude mice subcutaneously. Tumors were excised at the end of the experiment. (F) H226 cells infected with sgTRIM26 or control sgRNA (NC) lentivirus were injected into nude mice subcutaneously. (G-H) Tumor weights were then measured at the end of each experiment. mean±SD (n=6). ***,* P*< 0.001, versus control group. (I) Tumor samples from the xenografts were subjected to IB analyses for TRIM26 and PBX1with specific antibodies. (J) Tumors from E-F were subjected to IHC against specific proteins as indicated. (K) The hypothesis of TRIM26 promotes NSCLC by inducing PBX1 degradation via the ubiquitin proteasome pathway.

Next, we analyzed the expression profiles of TRIM26 in NSCLC patients and its biological activity *in vivo* on NSCLC tumor growth. We first examined TRIM26 expression in NSCLC tissues. As shown in Fig. 8A, TRIM26 was strikingly highly expressed in NSCLC tissues compared with the normal control lung tissues. The score of the staining in TRIM26 expression was also significantly upregulated (Fig. 8B). Next, we collected NSCLC tissues for IB assays and found that TRIM26 was also highly expressed (Fig. 8C), negatively associated with the PBX1 expression (Fig. 1). Moreover, different from PBX1 as a favor predictor in NSCLC patients, TRIM26 was negatively associated with the overall survival of NSCLC patients (Fig. 8D). The data, along with the cell proliferation assays (Fig. 6), suggest that TRIM26 could be an oncogenic and promotes NSCLC malignant development. To further solidify this conclusion, TRIM26 was overexpressed into or knocked out from NSCLC cell lines followed by xenograft growth evaluation in nude mice. The results showed that overexpression of TRIM26 markedly promoted the tumor growth (Fig. 8E and G) while knockout of TRIM26 suppressed NSCLC tumor growth in mice (Fig. 8F and H). Moreover, the IB analyses on tumor tissues from xenografts showed that overexpression of TRIM26 decreased PBX1 and RNF6, while knockout of TRIM26 led to upregulation of PBX1 and RNF6 (Fig. 8I and J). Taken all together, these results concluded that TRIM26 promotes NSCLC tumor growth by suppressing PBX1 via the ubiquitin-proteasomal pathway (Fig. 8K).

## Discussion

The present study identifies TRIM26 is a putative ubiquitin ligase of PBX1. TRIM26 interacts with PBX1 and mediates its K48-linked polyubiquitination and proteasomal degradation therefore inhibiting PBX1 transcriptional activity and promotes NSCLC cell proliferation and tumor survival.

TRIM26 is a member of the tripartite motif (TRIM) family E3 ligases containing the TRIM domain. Currently, most studies on TRIM26 are focused on its involvement in modulation of innate antiviral responses^15^. There are several studies on cancers but present contradicted conclusion. On one side, TRIM26 is reported as a tumor suppressor and its overexpression inhibits the growth of tumors including hepatocellular carcinoma^16^, and thyroid cancer^17^. On the other hand, it is also found that TRIM26 might promote carcinogenesis of bladder cancer by activating AKT/GSK3β signaling^18^ and is involved in maintenance of glioblastoma stem cells by stabilizing SOX2, a key transcription factor in cancer stem cells^19^. In the present study, we added that TRIM26 also promotes NSCLC and it clearly demonstrated that TRIM26 is highly expressed in NSCLC cells and promotes NSCLC cell proliferation, migration and tumor growth. In the mechanistic study, we found that although TRIM26 fails to modulate PTEN, it induces degradation of PBX1 in NSCLC cells as a ubiquitin E3 ligase. The AP-MS/MS and subsequent reciprocal IP/IB assays demonstrated that TRIM26 binds to PBX1. TRIM26 mediates PBX1 for K48-linked polyubiquitination, however, when the RING domain is deleted, TRIM26 is inactivated and it fails to induce PBX1 degradation. Therefore, TRIM26 as an E3 ligase mediates PBX1 ubiquitination and degradation.

Moreover, PBX1 is also an interesting gene/protein. Previous studies have revealed that PBX1 acts as an oncogene involved in various cancers, including leukemia^6^, gastric cancer^4^, prostate cancer^8^, myeloma^20^. Our present study confirmed this finding and found that PBX1 alone inhibits NSCLC cell proliferation, colony formation, and survival. Moreover, the public data analysis on 1200 NSCLC patients reveals PBX1 is positively associated with overall survival of NSCLC patients. It is reported that PBX1 transcription is modulated by the transcription factor/enhancers such as NICD3/CSL^21^, NANOG^22^ or miRNA^23^. Our previous study found that the PBX1 protein can be regulated via the ubiquitin-proteasomal pathway and could be stabilized by the deubiquitinase USP9x^8^. In the present study, we further found that PBX1 undergoes K48-linked polyubiquitination and proteasomal degradation under the direction of the ubiquitin ligase TRIM26, a promoter of NSCLC cell survival.

However, PBX1 is a transcription factor and how it suppresses NSCLC is unknown. Several downstream signaling pathways are reported, in which PBX1 acts as a pioneer transcription factor to mediate the binding of FoxC1 to ZEB2 promoter in oesophageal cancer cells^24^, or to promote 13‐cis retinoic acid‐mediated differentiation in neuroblastoma^25^. We found that PBX1 acts as a transcription factor to upregulate RNF6 in leukemia cells^6^. In the present study, we found that both PBX1 and RNF6 are downregulated in NSCLC, suggesting the PBX1 and RNF6 might suppress NSCLC. This finding is consistent with a previous that PBX1 inhibits NSCLC cell proliferation 26 and RNF6 might act as a tumor suppressor in esophageal cancer 27. Although a recent study reported that RNF6 is highly expressed in NSCLC tissues based on a database at Kaplan-Meier Plotter (http://kmplot.com/) but the plots could not be reproduced^28^. In the same study, the RING domain-deleting RNF6 remains the same size as the wild type in the IB assay^28^, this result is impossible because the RING domain is composed of 44 amino acids and the molecular weight is around 5 kDa, which could be clearly distinguished 9,29.In the present study, we solidified the conclusion that PBX1/RNF6 was downregulated in NSCLC tissues and suppress cell proliferation. However, the molecular mechanism underlying that RNF6 inhibits NSCLC should be further investigated.

In summary, the present study demonstrates TRIM26 is a first reported ubiquitin ligase of PBX1 and elucidates the detailed mechanism under TRIM26 promotes NSCLC cell proliferation and tumor growth by targeting PBX1. This study also highlights TRIM26/PBX1 could be a potential therapeutic target of NSCLC.

## Abbreviations

CHX: cycloheximide
EdU: 5-ethynyl-2'-deoxyuridine
GAPDH: glyceraldehyde-3-phosphate dehydrogenase
IB: immunoblotting
IF: Immunofluorescence
IHC: Immunohistochemistry
IP: immunoprecipitation
MTT: 3-(4,5)-dimethylthiahiazo(-z-y1)-3,5-di-phenytetrazoliumromide
NSCLC: non-small cell lung cancer
PBX1: PBX Homeobox 1
Rb: retinoblastoma gene
RNF6: Ring finger protein 6
RT-qPCR: quantitative reverse transcription PCR
SDS: Sodium dodecyl sulfate
sgRNA: single guide RNA
shRNA: short hairpin RNA
siRNA: small interfering RNA
TRIM26: tripartite motif containing 26
Ub: ubiquitination (ubiquitin)
